# Assessment of the Impact of Statin Use to Predict All‐Cause Mortality in Patients With Critical Cerebrovascular Disease: A Retrospective Cohort Study From the MIMIC‐IV Database

**DOI:** 10.1111/cns.70542

**Published:** 2025-07-27

**Authors:** Dong Tang, Zheng Huang, Shifu Li, Fenghua Chen

**Affiliations:** ^1^ Department of Neurosurgery Xiangya Hospital, Central South University Changsha China; ^2^ Cerebrovascular Diseases Research Center Xiangya Hospital, Central South University Changsha China; ^3^ National Clinical Research Center for Geriatric Disorders Xiangya Hospital, Central South University Changsha China

**Keywords:** all‐cause mortality, hemorrhagic stroke, intensive care unit, ischemic stroke, MIMIC‐IV database, statin

## Abstract

**Background:**

The impact of statin therapy on short‐term mortality among critically ill patients with hemorrhagic stroke or ischemic stroke remains uncertain. We investigated associations between statin use and ICU and hospital mortality in this patient population.

**Methods:**

We conducted a retrospective cohort study using the MIMIC‐IV database, including 6918 patients (2960 HS and 3958 IS) after applying strict exclusion criteria. Statin use was assessed by type, dose (standard vs. high), and initiation timing (pre‐ICU vs. post‐ICU). Survival outcomes were evaluated using Kaplan–Meier analysis and multivariable Cox regression models, landmark analyses, and Fine–Gray competing‐risk models, with propensity score matching to adjust for confounding factors.

**Results:**

Statin use significantly reduced ICU mortality at 30 days in HS (HR = 0.59, 95% CI: 0.41–0.87) and IS (HR = 0.45, 95% CI: 0.32–0.64) cohorts. Atorvastatin and simvastatin showed pronounced protective effects, independent of dose intensity. Post‐ICU initiation of statins conferred greater benefit compared with pre‐ICU initiation, especially in HS patients. Shorter statin treatment duration (≥ 3 days) sufficiently captured beneficial effects. Patients with hyperlipidemia demonstrated enhanced mortality benefit.

**Conclusion:**

Statin use is associated with significantly lower short‐term mortality in critically ill stroke patients, supporting tailored strategies for optimal statin initiation and duration.

## Graphical abstract

Statin use in critically ill stroke patients is associated with reduced short‐term ICU and hospital mortality, particularly with post‐ICU initiation and at least 3 days of treatment. Atorvastatin and simvastatin showed the strongest protective effects. These findings support tailored statin strategies in acute stroke care.

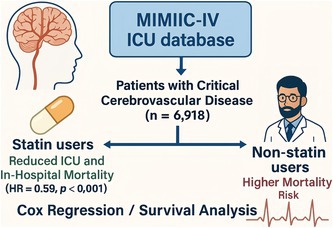

## 1 Introduction

Cerebrovascular diseases, including hemorrhagic stroke (HS) and ischemic stroke (IS), remain leading causes of mortality and disability worldwide. According to the Global Burden of Disease (GBD) Study, stroke is the second most common cause of death globally, contributing to significant healthcare costs and long‐term disability [1, 2]. The incidence of IS far exceeds that of HS, yet the latter is associated with higher mortality and worse functional outcomes [3]. Among critically ill patients admitted to intensive care units (ICUs), cerebrovascular diseases present unique challenges, with high risks of neurological deterioration, systemic complications, and poor prognosis. These patients often experience life‐threatening conditions such as malignant cerebral edema, intracranial hypertension, or refractory seizures [4, 5]. Moreover, systemic complications, including respiratory failure, cardiac dysfunction, and infections, further contribute to increased mortality risk. Given these challenges, optimizing medical management, including pharmacological interventions, is crucial for improving patient outcomes [6, 7]. Among various therapeutic agents, statins have emerged as potential neuroprotective agents beyond their well‐established lipid‐lowering effects.

Statins are a class of lipid‐lowering agents that function primarily by inhibiting 3‐hydroxy‐3‐methylglutaryl‐coenzyme A (HMG‐CoA) reductase, thereby reducing cholesterol synthesis [8]. By lowering low‐density lipoprotein (LDL) cholesterol levels, statins play a central role in cardiovascular disease prevention. Additionally, statins exert pleiotropic effects, including anti‐inflammatory, antioxidative, endothelial‐protective, and immunomodulatory properties, which may contribute to improved outcomes in cerebrovascular diseases [9, 10]. Beyond their cholesterol‐lowering effects, statins have demonstrated multiple beneficial properties that could be particularly relevant in critically ill cerebrovascular patients. Preclinical and clinical studies suggest that statins: reduce neuroinflammation by inhibiting pro‐inflammatory cytokines such as tumor necrosis factor‐alpha (TNF‐α) and interleukins [11, 12]; enhance endothelial function and stabilize atherosclerotic plaques, reducing the risk of further vascular events [13]; promote neuroprotection through increased nitric oxide (NO) production and modulation of cerebral blood flow [14, 15]; influence immune response and thrombosis by modulating platelet aggregation and coagulation pathways [16].

The role of statins in acute stroke has been widely studied in non‐ICU settings, demonstrating benefits in functional recovery and secondary prevention [17, 18]. However, their impact on critically ill patients with cerebrovascular disease remains an area of ongoing investigation. In IS, early statin use has been associated with reduced mortality and improved functional outcomes, possibly due to its anti‐inflammatory and neuroprotective properties. Some retrospective studies suggest that prior statin use may reduce ICU mortality in stroke patients, though results remain inconclusive due to potential confounding factors [19]. Statins have shown potential neuroprotective effects in intracerebral hemorrhage (ICH), including reduced perihematomal edema, lower mortality rates, and improved functional outcomes [20]. However, their use in HS remains controversial. Some studies suggest statins may increase the risk of ICH recurrence or hematoma expansion [21, 22], while others report no significant impact on ICH recurrence [23]. Additionally, the potential interaction between statins and anticoagulant/antiplatelet therapies in HS patients raises safety concerns that warrant further investigation. While some observational studies have suggested a neutral or even protective effect of statins on hematoma expansion [24], others have reported increased rebleeding rates [25]. Understanding this risk is essential for optimizing the therapeutic approach in critically ill patients.

Given the complexities of critical cerebrovascular disease and the potential impact of statin therapy, a comprehensive analysis using a high‐quality clinical dataset is necessary. The Medical Information Mart for Intensive Care (MIMIC‐IV) database provides a valuable resource for conducting real‐world retrospective studies, offering detailed patient records, medication data, and clinical outcomes. This study aims to evaluate the impact of statin use on all‐cause mortality in critically ill cerebrovascular patients, providing new insights into its potential benefits and risks in this high‐risk population.

## 2 Method

### 2.1 Study Population

This study performs a retrospective analysis utilizing publicly available data from the MIMIC‐IV database. This dataset encompasses clinical records of critically ill patients admitted to Beth Israel Deaconess Medical Center between 2008 and 2019. Data access was granted following the completion of the mandatory training course provided by the National Institutes of Health (NIH) and successful certification through the Collaborative Institutional Training Initiative (CITI) program (certification number: 68889220). To ensure patient confidentiality, all personal identifiers in the database have been de‐identified, allowing for an exemption from the requirement for informed consent.

**FIGURE 1:**
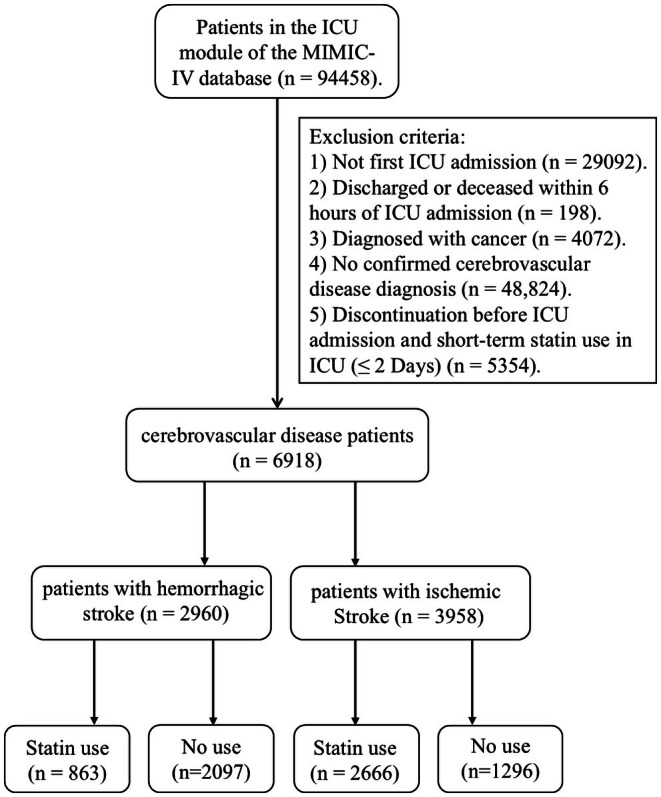
Flowchart of select study population from the MIMIC‐IV database.

We analyzed a total of 94,458 patients aged 18 years and older from the database. Patients were excluded based on the following criteria: (1) Those who had multiple ICU admissions, retaining only the first admission (*n* = 29,092); (2) Those with an ICU stay of less than 6 h (*n* = 198); (3) Patients diagnosed with cancer (*n* = 4072) (4) Those without a diagnosis of cerebrovascular disease, as defined by specific International Classification of Diseases (ICD) codes (see Supporting Information S1) (*n* = 48,824) (5) Patients who discontinued statin use before ICU admission or had short‐term statin use (≤ 2 days) in the ICU (*n* = 5354). After applying these criteria, a total of 2960 patients with HS and 3958 patients with IS were included in the final analysis (Figure 1).

### 2.2 Data Collection

All data extraction, statistical analyses, and visualizations were performed using R Studio (version 4.3.1). The extracted variables encompassed the following categories: (1) demographic information: age, sex, and race; (2) vital signs: heart rate, systolic blood pressure, diastolic blood pressure, mean blood pressure; (3) comorbid conditions: chronic kidney disease, liver disease, hypertension, atrial fibrillation, chronic obstructive pulmonary disease, diabetes mellitus, congestive heart failure, hyperlipidemia; (4) laboratory indicators: RBC, WBC, platelet, hemoglobin, hematocrit, anion gap, bicarbonate, blood urea nitrogen (BUN), calcium, chloride, creatinine, glucose, sodium, potassium, INR, PT, PTT; (5) disease severity score: Sequential Organ Failure Assessment (SOFA), Simplified Acute Physiology Score II (SAPSII), Oxford Acute Severity of Illness Score (OASIS), Glasgow coma scale (GCS); (6) treatment: continuous renal replacement therapy, ventilation, antiplatelet drug, anticoagulant drug, vasoactive; (6) length of stay (LOS) and patient outcomes. All laboratory measurements and illness severity scores were obtained within the first 24 h of ICU admission. To reduce potential bias, variables with missing data exceeding 20% were excluded from the analysis. For variables with a low proportion of missing data, missing values were imputed using an iterative estimation method based on the random forest algorithm (missForest) [26].

### 2.3 Patterns of Statin Use

Patients were classified into the statin group and the non‐statin group based on statin use. Additionally, we assessed the association between different types of statins and all‐cause mortality, including four commonly prescribed statins: atorvastatin, pravastatin, rosuvastatin calcium, and simvastatin. To further explore the impact of statin dosage on mortality in critically ill patients with cerebrovascular disease, statin use was stratified into standard‐dose and high‐dose groups. High‐dose statin therapy was defined as exceeding the following daily dosages: atorvastatin 40 mg, simvastatin 40 mg, pravastatin 40 mg, and rosuvastatin calcium 20 mg [27]. Patients who received multiple types or varying doses of statins during their ICU stay were excluded to ensure consistency in exposure assessment. Furthermore, statin use was categorized into pre‐admission statin therapy and post‐ICU initiation. Pre‐admission statin use was defined as the continuation of statin therapy that began prior to ICU admission and persisted for more than 2 days after ICU admission.

### 2.4 Outcome Indicators

The primary outcome of this study was all‐cause mortality during the ICU stay and hospitalization among critically ill patients with cerebrovascular disease. Secondary outcomes included 30‐ and 90‐day mortality, as well as the length of ICU stay and overall hospital stay.

### 2.5 Statistical Analysis

Continuous variables were first assessed for normality using the Shapiro–Wilk test. Variables following a normal distribution are presented as mean ± standard deviation and were compared between groups using Student's *t*‐test. Non‐normally distributed variables are summarized as median with interquartile range (IQR) and were compared using the Mann–Whitney U test. Categorical variables are reported as counts (percentages) and compared between groups using the chi‐square test or Fisher's exact test, as appropriate.

Survival probabilities at 30‐ and 90‐day intervals were estimated using Kaplan–Meier methods, with statistical significance determined via log‐rank tests. Multivariable Cox proportional hazards regression models were employed to estimate the association between statin use and all‐cause ICU mortality, with results reported as adjusted hazard ratios (aHR) and 95% confidence intervals (CI). Seven sequential models were constructed: crude model (unadjusted), model 1 (demographics‐adjusted), model 2 (vital signs‐adjusted), model 3 (comorbidities‐adjusted), model 4 (laboratory indicators‐adjusted), model 5 (disease severity‐adjusted), and model 6 (fully adjusted). The non‐statin cohort was used as the reference group.

To mitigate immortal time bias and examine the time‐dependent effect of statin therapy, we conducted landmark analyses by excluding patients who died within the first 2, 3, 5, or 7 days following ICU admission. Patients who received statins beyond each respective landmark time point (≥ 2, ≥ 3, ≥ 5, or ≥ 7 days) were compared to those who received no statins, and multivariable Cox regression models were then re‐estimated within each landmark‐defined cohort.

Given that ICU discharge represents a competing risk for in‐ICU death, we further applied the Fine–Gray subdistribution hazard model to account for the competing risk of being discharged alive. This analysis was implemented using the cmprsk package in R to calculate subdistribution hazard ratios (SHRs), providing a more accurate estimation of the cumulative incidence of death in the presence of competing events. To address residual confounding, propensity score matching (PSM) was performed using the MatchIt package (version 4.7.1), applying 1:1 nearest‐neighbor matching without replacement and a caliper width of 0.2 standard deviations of the logit of the propensity score. Covariate balance before and after matching was assessed using standardized mean differences (SMDs), with SMD < 0.1 considered acceptable. All analyses were conducted using R software (version 4.4.0). Two‐tailed *p* values < 0.05 were considered statistically significant.

## 3 Results

### 3.1 Cohort Characteristics

As illustrated in Figure 1, a total of 6918 patients were included in this study, comprising 2960 patients with HS and 3958 patients with IS. The median age of patients with HS was 67.14 years, with 1449 being female. Among them, 499 patients died during their ICU stay, and 645 died during hospitalization. For patients with IS, the median age was 71.47 years, with 2128 females. Within this group, ICU mortality was recorded in 328 patients, while 503 patients died during hospitalization (Table 1). In the cohort of patients with severe cerebrovascular diseases, statin users exhibited significantly advanced age (*p* < 0.001) and higher prevalence of chronic kidney disease (*p* < 0.001), diabetes mellitus (*p* < 0.001), congestive heart failure (*p* < 0.001), and hyperlipidemia (*p* < 0.001). They also demonstrated lower serum sodium levels (*p* < 0.001), reduced anion gap (p < 0.001), and greater likelihood of receiving antiplatelet/anticoagulant therapy (*p* < 0.001). After landmark analyses and propensity score matching (PSM), the final analysis included 956 HS cases (comprising 478 statin users and 478 non‐users) and 1186 IS cases (including 593 statin users and 593 non‐users). All baseline characteristics achieved optimal balance between groups, with standardized mean differences (SMD) < 0.1 and all variables showing *p* < 0.05 (Table S1).

**TABLE 1:** Baseline characteristics of patients with hemorrhagic stroke and ischemic stroke.

Variables	Hemorrhagic stroke				Ischemic stroke			
	Total (*n* = 2960)	No use (*n* = 2097)	Statin use (*n* = 863)	*p*	Total (*n* = 3958)	No use (*n* = 1292)	Statin use (*n* = 2666)	*p*
**Demographics**								
Age, years	67.14 ± 16.01	65.56 ± 16.86	70.99 ± 12.95	< 0.0001	71.47 ± 14.32	68.84 ± 17.56	72.75 ± 12.25	< 0.0001
Sex, female, *n* (%)	1449 (48.95)	1057 (50.41)	392 (45.42)	0.02	2128 (53.76)	607 (46.98)	1521 (57.05)	< 0.0001
Race				< 0.01				0.03
Other, *n* (%)	1317 (44.49)	966 (46.07)	351 (40.67)		1843 (46.56)	634 (49.07)	1209 (45.35)	
White, *n* (%)	1643 (55.51)	1131 (53.93)	512 (59.33)		2115 (53.44)	658 (50.93)	1457 (54.65)	
**Vital signs**								
HR mean, beats/min	79.64 ± 14.02	80.27 ± 14.30	78.13 ± 13.19	< 0.0001	81.19 ± 15.00	83.14 ± 16.65	80.24 ± 14.03	< 0.0001
SBP mean, mmHg	128.85 ± 14.50	127.78 ± 14.53	131.47 ± 14.10	< 0.0001	125.50 ± 18.52	123.38 ± 17.86	126.53 ± 18.76	< 0.0001
DBP mean, mmHg	67.44 ± 11.11	67.12 ± 10.70	68.22 ± 12.01	0.02	67.18 ± 12.81	67.19 ± 12.39	67.18 ± 13.01	0.99
MBP mean, mmHg	84.79 ± 10.37	84.26 ± 10.04	86.07 ± 11.04	< 0.0001	84.17 ± 12.72	83.53 ± 12.22	84.48 ± 12.94	0.02
**Comorbidity**								
CKD, *n* (%)	294 (9.93)	159 (7.58)	135 (15.64)	< 0.0001	951 (24.03)	221 (17.11)	730 (27.38)	< 0.0001
LD, *n* (%)	16 (0.54)	11 (0.52)	5 (0.58)	1.00	30 (0.76)	11 (0.85)	19 (0.71)	0.78
HTN, *n* (%)	1770 (59.80)	1244 (59.32)	526 (60.95)	0.44	1845 (46.61)	592 (45.82)	1253 (47.00)	0.51
AF, *n* (%)	541 (18.28)	330 (15.74)	211 (24.45)	< 0.0001	832 (21.02)	266 (20.59)	566 (21.23)	0.67
COPD, *n* (%)	99 (3.34)	54 (2.58)	45 (5.21)	< 0.001	466 (11.77)	121 (9.37)	345 (12.94)	< 0.01
DM, *n* (%)	653 (22.06)	344 (16.40)	309 (35.81)	< 0.0001	1492 (37.70)	333 (25.77)	1159 (43.47)	< 0.0001
CHF, *n* (%)	333 (11.25)	160 (7.63)	173 (20.05)	< 0.0001	1182 (29.86)	283 (21.90)	899 (33.72)	< 0.0001
HLP, *n* (%)	1157 (39.09)	568 (27.09)	589 (68.25)	< 0.0001	2318 (58.56)	446 (34.52)	1872 (70.22)	< 0.0001
**Laboratory indicators**								
RBC (10^12^/L)	4.03 ± 0.60	4.03 ± 0.60	4.05 ± 0.60	0.38	3.73 ± 0.68	3.72 ± 0.68	3.74 ± 0.68	0.44
WBC (10^9^/L)	11.92 ± 8.11	12.10 ± 9.19	11.48 ± 4.48	0.01	12.19 ± 7.22	12.87 ± 9.65	11.86 ± 5.65	< 0.001
Platelet (10^9^/L)	215.90 ± 68.47	215.56 ± 69.03	216.72 ± 67.12	0.67	206.93 ± 82.40	206.36 ± 88.17	207.20 ± 79.47	0.77
Hemoglobin (g/L)	12.15 ± 1.68	12.16 ± 1.67	12.12 ± 1.70	0.57	11.00 ± 1.96	10.96 ± 1.95	11.02 ± 1.97	0.35
Hematocrit (%)	38.96 ± 5.55	38.90 ± 5.54	39.09 ± 5.58	0.41	36.34 ± 6.42	36.41 ± 6.58	36.31 ± 6.35	0.66
Aniongap (mmol/L)	15.82 ± 3.76	15.99 ± 3.72	15.42 ± 3.81	< 0.001	15.65 ± 4.63	16.23 ± 4.88	15.37 ± 4.48	< 0.0001
Bicarbonate (mmol/L)	24.21 ± 3.21	24.09 ± 3.14	24.52 ± 3.36	< 0.01	23.49 ± 3.45	23.20 ± 3.68	23.64 ± 3.33	< 0.001
BUN (mg/dl)	20.24 ± 13.92	19.70 ± 13.76	21.55 ± 14.24	< 0.01	27.14 ± 21.80	27.16 ± 23.86	27.13 ± 20.74	0.96
Calcium (mg/dL)	8.91 ± 0.63	8.89 ± 0.62	8.95 ± 0.65	0.03	8.79 ± 0.69	8.72 ± 0.73	8.82 ± 0.67	< 0.001
Chloride (mmol/L)	106.26 ± 5.92	106.54 ± 6.10	105.58 ± 5.39	< 0.0001	105.35 ± 5.89	105.94 ± 6.56	105.07 ± 5.51	< 0.0001
Creatinine (mg/dL)	1.13 ± 0.98	1.10 ± 0.89	1.21 ± 1.18	0.01	1.47 ± 1.45	1.38 ± 1.25	1.52 ± 1.54	< 0.01
Glucose (mmol/L)	162.37 ± 79.63	160.80 ± 82.06	166.18 ± 73.29	0.08	166.63 ± 93.16	163.83 ± 78.28	167.99 ± 99.56	0.15
Sodium (mmol/L)	141.48 ± 5.22	141.75 ± 5.40	140.84 ± 4.71	< 0.0001	140.26 ± 5.07	140.84 ± 5.77	139.98 ± 4.68	< 0.0001
Potassium (mmol/L)	4.28 ± 0.75	4.26 ± 0.75	4.31 ± 0.74	0.16	4.55 ± 0.87	4.51 ± 0.84	4.57 ± 0.88	0.02
INR	1.33 ± 0.94	1.34 ± 1.08	1.29 ± 0.47	0.10	1.47 ± 0.85	1.53 ± 1.02	1.45 ± 0.75	0.01
PT (s)	14.50 ± 8.70	14.64 ± 9.87	14.15 ± 4.79	0.07	16.31 ± 10.33	17.01 ± 12.53	15.97 ± 9.06	< 0.01
PTT (s)	33.38 ± 18.51	33.38 ± 18.84	33.37 ± 17.68	0.99	44.24 ± 30.57	42.47 ± 29.13	45.09 ± 31.21	< 0.01
**Disease severity score**								
SOFA	3.35 ± 2.67	3.41 ± 2.79	3.20 ± 2.36	0.03	4.18 ± 3.21	4.50 ± 3.58	4.02 ± 3.00	< 0.0001
SAPSII	33.24 ± 12.51	33.25 ± 12.83	33.20 ± 11.70	0.92	35.96 ± 13.00	36.64 ± 14.69	35.64 ± 12.09	0.03
OASIS	31.66 ± 8.11	31.82 ± 8.20	31.28 ± 7.88	0.09	31.56 ± 8.39	32.58 ± 8.94	31.06 ± 8.06	< 0.0001
GCS	12.65 ± 3.24	12.65 ± 3.35	12.65 ± 2.95	0.98	13.27 ± 2.81	13.00 ± 3.10	13.40 ± 2.65	< 0.0001
Treatment								
CRRT, *n* (%)	33 (1.11)	26 (1.24)	7 (0.81)	0.41	74 (1.87)	32 (2.48)	42 (1.58)	0.07
Ventilation, *n* (%)	1237 (41.79)	937 (44.68)	300 (34.76)	< 0.0001	1224 (30.92)	449 (34.75)	775 (29.07)	< 0.001
Antiplatelet drug, *n* (%)	841 (28.41)	366 (17.45)	475 (55.04)	< 0.0001	2779 (70.21)	596 (46.13)	2183 (81.88)	< 0.0001
Anticoagulant drug, *n* (%)	2168 (73.24)	1400 (66.76)	768 (88.99)	< 0.0001	3243 (81.94)	1018 (78.79)	2225 (83.46)	< 0.001
Vasoactive drug, *n* (%)	565 (19.09)	397 (18.93)	168 (19.47)	0.78	998 (25.21)	337 (26.08)	661 (24.79)	0.40
**Length of stay (LOS)**								
LOS in hospital	12.79 ± 15.18	11.98 ± 15.99	14.77 ± 12.78	< 0.0001	12.73 ± 13.34	12.39 ± 14.69	12.90 ± 12.64	0.28
LOS in ICU	6.82 ± 8.00	6.54 ± 7.98	7.52 ± 8.02	< 0.01	4.93 ± 6.28	5.25 ± 6.59	4.78 ± 6.11	0.03
**Outcomes**								
ICU death, *n* (%)	499 (16.86)	445 (21.22)	54 (6.26)	< 0.0001	328 (8.29)	123 (4.61)	205 (15.87)	< 0.0001
In‐hospital death, *n* (%)	645 (21.79)	560 (26.70)	85 (9.85)	< 0.0001	503 (12.71)	216 (8.10)	287 (22.21)	< 0.0001
30‐day mortality, *n* (%)	517 (17.47)	464 (22.13)	53 (6.14)	< 0.0001	336 (8.49)	211 (16.34)	125 (4.69)	< 0.0001
90‐day mortality, *n* (%)	524 (17.70)	468 (22.32)	56 (6.49)	< 0.0001	340 (8.59)	213 (16.50)	127 (4.76)	< 0.0001

Abbreviations: AF, atrial fibrillation; CHF, congestive heart failure; CKD, chronic kidney disease; COPD, chronic obstructive pulmonary disease; CRRT, continuous renal replacement therapy; DBP, diastolic blood pressure; DM, diabetes mellitus; GCS, Glasgow coma scale; HLP, hyperlipidemia; HR, heart rate; HTN, hypertension; INR, international normalized ratio; LD, liver disease; MBP, mean blood pressure; OASIS, oxford acute severity of illness score; PT, prothrombin time; PTT, partial thromboplastin time. SOFA, sequential organ failure assessment; SAPSII, simplified acute physiology score; SBP, systolic blood pressure.

### 3.2 Statin Use and Clinical Outcomes

**FIGURE 2:**
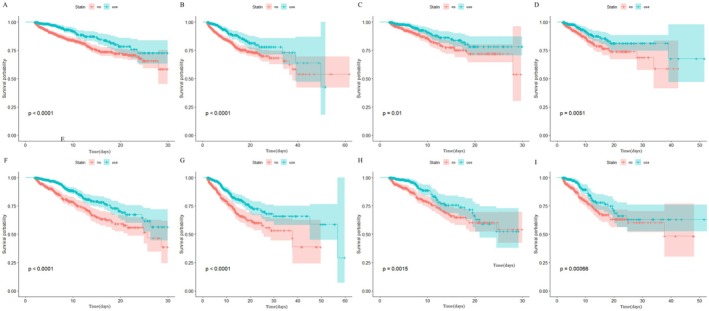
Kaplan–Meier survival curves of the non‐statin group and statin group for ICU mortality. (A) 30‐day ICU mortality of hemorrhagic stroke; (B) 90‐day ICU mortality of hemorrhagic stroke; (C) 30‐day ICU mortality of hemorrhagic stroke after PSM; (D) 90‐day ICU mortality of hemorrhagic stroke after PSM; (E) 30‐day ICU mortality of ischemic stroke; (F) 90‐day ICU mortality of ischemic stroke; (G) 30‐day ICU mortality of ischemic stroke after PSM; (H) 30‐day ICU mortality of ischemic stroke after PSM.

Survival outcomes were assessed using Kaplan–Meier analysis to evaluate differences in ICU and in‐hospital mortality between statin users and non‐users across different follow‐up periods in distinct stroke subtypes. In the original cohort, statin users demonstrated significantly lower ICU and in‐hospital mortality at both 30‐day and 90‐day intervals compared to non‐users (log‐rank *p* < 0.001; Figure 2). After landmark analyses and PSM, the Kaplan–Meier survival curves remained consistent with the original cohort findings (Figure 2 and Figure S1). To further investigate the association between statin use and mortality, we performed Cox proportional hazards regression analyses (Table 2). In the fully adjusted model (Model 6), statin use remained independently associated with a 41% and 42% reduction in 30‐day (HR 0.59, 95% CI: 0.41–0.87, *p* < 0.001) and 90‐day (HR 0.58, 95% CI: 0.40–0.86, *p* < 0.001) ICU mortality in HS patients, while IS patients exhibited a 55% risk reduction at both time points (30‐day HR 0.45, 95% CI: 0.32–0.64; 90‐day HR 0.45, 95% CI: 0.32–0.64; *p* < 0.001 for both). In the Fine–Gray model, statin therapy was associated with a 44% and 46% reduction in 30‐day (HR 0.56, 95% CI: 0.34–0.91, *p* = 0.019) and 90‐day (HR 0.54, 95% CI: 0.33–0.87, *p* = 0.014) ICU mortality risk in HS patients. Similarly, in IS patients, statin use correlated with a 56% risk reduction in 30‐day (HR 0.44, 95% CI: 0.31–0.65, *p* < 0.001) and 90‐day (HR 0.44, 95% CI: 0.30–0.64, *p* < 0.001) ICU mortality, respectively. Consistently, statin therapy significantly reduced in‐hospital mortality risk at 30‐day and 90‐day intervals in both HS and IS cohorts (Table S2).

**TABLE 2:** Association between the statin group and all‐cause ICU mortality on Landmark analyses.

Mortality		HS‐30‐day mortality		HS‐90‐day mortality		IS‐30‐day mortality		IS‐90‐day mortality	
	No use	HR 95% CI	*p*	HR 95% CI	*p*	HR 95% CI	*p*	HR 95% CI	*p*
CM	Ref	0.51 (0.37, 0.70)	< 0.0001	0.51 (0.38, 0.70)	< 0.0001	0.47 (0.36, 0.60)	< 0.0001	0.46 (0.35, 0.59)	< 0.0001
Model 1	Ref	0.44 (0.32, 0.61)	< 0.0001	0.44 (0.32, 0.60)	< 0.0001	0.39 (0.30, 0.51)	< 0.0001	0.38 (0.30, 0.50)	< 0.0001
Model 2	Ref	0.45 (0.33, 0.63)	< 0.0001	0.45 (0.33, 0.62)	< 0.0001	0.41 (0.31, 0.53)	< 0.0001	0.40 (0.31, 0.52)	< 0.0001
Model 3	Ref	0.39 (0.28, 0.55)	< 0.0001	0.39 (0.27, 0.55)	< 0.0001	0.36 (0.27, 0.47)	< 0.0001	0.35 (0.27, 0.47)	< 0.0001
Model 4	Ref	0.39 (0.28, 0.56)	< 0.0001	0.39 (0.27, 0.56)	< 0.0001	0.36 (0.26, 0.49)	< 0.0001	0.36 (0.26, 0.49)	< 0.0001
Model 5	Ref	0.40 (0.28, 0.58)	< 0.0001	0.40 (0.28, 0.57)	< 0.0001	0.37 (0.27, 0.52)	< 0.0001	0.37 (0.27, 0.52)	< 0.0001
Model 6	Ref	0.59 (0.41, 0.87)	0.010	0.58 (0.40, 0.86)	0.010	0.45 (0.32, 0.64)	< 0.0001	0.45 (0.32, 0.64)	< 0.0001
PSM	Ref	0.52 (0.32, 0.85)	0.010	0.52 (0.32, 0.84)	0.010	0.49 (0.33, 0.73)	< 0.001	0.49 (0.33, 0.73)	< 0.001
Fine‐gray	Ref	0.56 (0.34, 0.91)	0.019	0.54 (0.33, 0.87)	0.014	0.44 (0.31, 0.65)	< 0.0001	0.44 (0.30, 0.64)	< 0.0001

*Note:* Crudel model: no adjusted. Model 1: crudel model, age, sex, race. Model 2: model 1, HR, SBP, DBP, MBP. Model 3: model 2, CKD, LIV, HTN, AF, COPD, DM, CHF, HLP. Model 4: model 3, RBC, WBC, platelet, hemoglobin, hematocrit, anion gap, bicarbonate, BUN, calcium, chloride, creatinine, glucose, sodium, potassium, INR, PT, PTT. Model 5: model 4, SOFA, SAPSII, OASIS, GCS. Model 6: model 5, CRRT, ventilation, antiplatelet drug, anticoagulant drug, vasoactive drug. Abbreviations: CI, confidence interval; CM, crude model; HR, hazard ratio; HS, hemorrhagic stroke; IS, ischemic stroke.

To further assess the robustness of these findings and the potential impact of unmeasured confounding, we calculated E‐values for the association between statin use and ICU mortality. For the main effect in HS patients, the observed hazard ratio (HR) of 0.56 (95% CI: 0.34–0.91) corresponds to an E‐value of 2.53, indicating that an unmeasured confounder would need to be associated with both statin use and ICU mortality by a risk ratio of at least 2.53, beyond the measured covariates, to fully explain away the observed association.

### 3.3 Statin Pharmacologic Profiles in Relation to All‐Cause Mortality

We identified a total of 702 patients with HS and 1790 patients with IS who were prescribed a single type and dose of statin therapy. Among HS patients, the distribution of statin use was as follows: atorvastatin (*n* = 422), simvastatin (*n* = 167), pravastatin (*n* = 72), and rosuvastatin calcium (*n* = 41). For IS patients, the distribution included atorvastatin (*n* = 1395), simvastatin (*n* = 169), pravastatin (*n* = 99), and rosuvastatin calcium (*n* = 128). To investigate the effect of different types and doses of statins on mortality, we conducted a subgroup analysis stratified by statin subtype and dosage. Compared to nonusers, the use of atorvastatin and simvastatin was significantly associated with reduced risk of all‐cause ICU mortality at both 30 and 90 days in patients with either HS or IS. Specifically, atorvastatin was associated with a 49% reduction in HS‐30‐day mortality (HR 0.51, 95% CI 0.33–0.80, *p* = 0.004) and a 62% reduction in IS‐30‐day mortality (HR 0.35, 95% CI 0.26–0.49, *p* < 0.0001). Simvastatin showed similarly strong protective effects (HS‐30‐day HR 0.39, 95% CI 0.21–0.70, *p* = 0.002; IS‐30‐day HR 0.29, 95% CI 0.14–0.60, *p* = 0.001). Although pravastatin and rosuvastatin calcium also showed trends toward reduced mortality, these associations did not reach statistical significance in the HS subgroup, likely due to smaller sample sizes (e.g., Pravastatin HS‐30‐day HR 0.55, 95% CI 0.22–1.36, *p* = 0.19) (Table 3).

**TABLE 3:** The association between different statins and all‐cause ICU mortality.

Mortality	HS‐30‐day mortality		HS‐90‐day mortality		IS‐30‐day mortality		IS‐90‐day mortality	
	HR 95% CI	*p*	HR 95% CI	*p*	HR 95% CI	*p*	HR 95% CI	*p*
Non‐statin	Ref		Ref		Ref		Ref	
**Types of statins**								
Atorvastatin	0.51 (0.33, 0.80)	0.004	0.52 (0.34, 0.82)	0.004	0.35 (0.26, 0.49)	< 0.0001	0.38 (0.29, 0.49)	< 0.0001
Pravastatin	0.55 (0.22, 1.36)	0.19	0.5 (0.20, 1.25)	0.14	0.28 (0.11, 0.70)	0.01	0.28 (0.11, 0.70)	0.01
Rosuvastatin Calcium	0.83 (0.30, 2.29)	0.72	0.83 (0.30, 2.30)	0.73	0.38 (0.18, 0.81)	0.01	0.38 (0.18, 0.81)	0.01
Simvastatin	0.39 (0.21, 0.70)	0.002	0.41 (0.23, 0.73)	0.003	0.29 (0.14, 0.60)	0.001	0.29 (0.14, 0.61)	0.001
**Doses of statins**								
High Dose	0.5 (0.31, 0.81)	0.005	0.5 (0.31, 0.81)	0.005	0.34 (0.25, 0.47)	< 0.0001	0.34 (0.24, 0.47)	< 0.0001
Standard dose	0.48 (0.31, 0.75)	0.001	0.48 (0.31, 0.75)	0.001	0.34 (0.22, 0.55)	< 0.0001	0.34 (0.21, 0.54)	< 0.0001

In terms of dose stratification, both standard‐dose and high‐dose statins were associated with significantly reduced ICU mortality compared with non‐statin use. High‐dose statins were associated with a 50% reduction in HS‐30‐day mortality (HR 0.50, 95% CI 0.31–0.81, *p* = 0.005) and a 66% reduction in IS‐30‐day mortality (HR 0.34, 95% CI 0.25–0.47, *p* < 0.0001). Interestingly, the protective effects of standard doses were comparable to high doses, suggesting that optimal therapeutic benefit might be achievable without increasing the dose‐related risk of adverse events. Similar trends were observed for in‐hospital mortality risk (Table S3).

### 3.4 Association Between Prior Statin Use and All‐Cause Mortality

We further stratified statin use based on the timing of initiation. Among patients who initiated statin therapy prior to ICU admission and continued for more than 2 days after ICU entry, there were 148 HS patients and 619 IS patients. In contrast, those who began statin therapy only after ICU admission and maintained it for more than 2 days numbered 715 HS patients and 2047 IS patients. Among HS patients, pre‐ICU statin use was consistently associated with increased ICU mortality across multiple models, including multivariable adjustment (HR 3.76, 95% CI: 1.39–10.19, *p* = 0.01), propensity score matching (HR 2.08, 95% CI: 0.93–4.68, *p* = 0.08), and Fine–Gray competing risk models (HR 1.85, 95% CI: 0.83–4.14, *p* = 0.13). In IS patients, no significant differences in ICU mortality were observed between groups, suggesting a neutral effect of pre‐ICU statin use (Table 4). Similar trends were observed for hospital mortality, with elevated risk in HS patients with pre‐ICU statin use and no statistically significant association in IS patients (Table S4), reinforcing the potential role of baseline differences and stroke subtype in modifying statin‐related outcomes.

**TABLE 4:** The association between pre‐ICU statin and post‐ICU statin for all‐cause ICU mortality based on Landmark analyses.

Variable	HS‐30‐day mortality		HS‐90‐day mortality		IS‐30‐day mortality		IS‐90‐day mortality	
	HR 95% CI	*p*	HR 95% CI	*p*	HR 95% CI	*p*	HR 95% CI	*p*
**Model 1**								
Post‐icu statin	Ref		Ref		Ref		Ref	
Pre‐icu statin	2.24 (0.98, 5.14)	0.06	2.1 (0.91, 4.83)	0.08	1.34 (0.84, 2.15)	0.22	1.32 (0.82, 2.11)	0.25
**Model 2**								
Post‐icu statin	Ref		Ref		Ref		Ref	
Pre‐icu statin	3.76 (1.39, 10.19)	0.01	3.52 (1.29, 9.62)	0.01	1.50 (0.92, 2.42)	0.10	1.4 7 (0.91, 2.38)	0.11
**PSM**								
Post‐icu statin	Ref		Ref		Ref		Ref	
Pre‐icu statin	2.08 (0.93, 4.68)	0.08	2.33 (1.00, 5.39)	0.05	1.18 (0.79, 1.76)	0.40	1.19 (0.82, 1.73)	0.35
**Fine‐Gray**								
Post‐icu statin	Ref		Ref		Ref		Ref	
Pre‐icu statin	1.85 (0.83, 4.14)	0.13	1.78 (0.82, 3.85)	0.14	1.46 (0.77, 2.76)	0.25	1.42 (0.76, 2.66)	0.28

*Note:* Model 1: crude model, Age, Sex, Race, HR, SBP, DBP, MBP, CKD, LIV, HTN, AF, COPD, DM, CHF, HLP; RBC, WBC, platelet, hemoglobin, hematocrit, anion gap, bicarbonate. Model 2: model 1, BUN, calcium, chloride, creatinine, glucose, sodium, potassium, INR, PT, PTT, SOFA, SAPSII, OASIS, GCS, CRRT, ventilation, antiplatelet drug, anticoagulant drug, vasoactive drug. Abbreviations: CI, confidence interval; HR, hazard ratio.

### 3.5 Sensitivity Analysis Stratified by Duration of Statin Use (≥ 3, ≥ 5, and ≥ 7 Days)

We further performed sensitivity analyses stratifying statin therapy duration (≥ 3, ≥ 5, and ≥ 7 days) compared to patients without statin use. Landmark analyses combined with multiple modeling approaches (including multivariable adjustment, PSM, and Fine–Gray competing risk analyses) were implemented to robustly assess the associations between statin exposure duration and clinical outcomes. As shown in Table 5, among HS patients, statin use for ≥ 3 days consistently demonstrated significant reductions in 30‐day ICU mortality (PSM HR 0.50, 95% CI: 0.28–0.90, *p* = 0.02; Fine–Gray HR 0.56, 95% CI: 0.34–0.92, *p* = 0.02). However, extending statin duration criteria to ≥ 5 or ≥ 7 days resulted in a weakened association and loss of statistical significance in both PSM and Fine–Gray models, indicating that a shorter duration (≥ 3 days) of exposure may be sufficient to capture beneficial pharmacodynamic effects, and longer durations may not yield further incremental survival benefits in HS patients. In IS patients, statin use of ≥ 3 and ≥ 5 days remained significantly associated with lower ICU mortality in most analyses. Nevertheless, the protective effect observed for ≥ 7 days statin use was less consistent and mostly not statistically significant in adjusted models (Model 2, PSM, and Fine–Gray analyses). These findings suggest a diminishing incremental benefit of prolonged statin exposure beyond 5 days in IS patients. Similar patterns were consistently observed for hospital mortality (Table S5).

**TABLE 5:** Association between duration of statin use (≥ 3, ≥ 5, and ≥ 7 Days) and all‐cause ICU mortality based on landmark analyses.

Variable	HS‐30‐day mortality		HS‐90‐day mortality		IS‐30‐day mortality		IS‐90‐day mortality	
	HR 95% CI	*p*	HR 95% CI	P	HR 95% CI	*p*	HR 95% CI	*p*
No use	Ref		Ref		Ref		Ref	
**Model 1**								
≥ 3 days	0.39 (0.25, 0.61)	< 0.01	0.39 (0.25, 0.61)	< 0.01	0.40 (0.28, 0.57)	< 0.01	0.39 (0.27, 0.55)	< 0.01
≥ 5 days	0.53 (0.33, 0.88)	0.01	0.53 (0.32, 0.87)	0.01	0.49 (0.32, 0.73)	< 0.01	0.56 (0.35, 0.89)	0.01
≥ 7 days	0.49 (0.27, 0.88)	0.02	0.48 (0.26, 0.87)	0.02	0.58 (0.36, 0.93)	0.02	0.56 (0.35, 0.90)	0.02
**Model 2**								
≥ 3 days	0.49 (0.31, 0.80)	< 0.01	0.49 (0.30, 0.79)	0.003	0.54 (0.36, 0.80)	< 0.01	0.52 (0.35, 0.78)	< 0.01
≥ 5 days	0.63 (0.37, 1.08)	0.09	0.62 (0.36, 1.07)	0.08	0.47 (0.31, 0.71)	< 0.01	0.54 (0.34, 0.85)	0.01
≥ 7 days	0.57 (0.30, 1.09)	0.09	0.55 (0.29, 1.06)	0.07	0.73 (0.42, 1.28)	0.27	0.69 (0.40, 1.21)	0.20
**PSM**								
≥ 3 days	0.5 (0.28, 0.90)	0.02	0.5 (0.28, 0.90)	0.02	0.68 (0.42, 1.11)	0.12	0.65 (0.40, 1.07)	0.09
≥ 5 days	0.47 (0.24, 0.94)	0.03	0.47 (0.24, 0.94)	0.03	0.61 (0.34, 1.08)	0.09	0.58 (0.32, 1.05)	0.07
≥ 7 days	0.54 (0.20, 1.44)	0.22	0.54 (0.20, 1.44)	0.22	0.65 (0.31, 1.37)	0.26	0.61 (0.29, 1.30)	0.20
**Fine–gray**								
≥ 3 days	0.56 (0.34, 0.92)	0.02	0.58 (0.36, 0.95)	0.03	0.48 (0.29, 0.79)	0.004	0.51 (0.31, 0.84)	< 0.01
≥ 5 days	0.67 (0.39, 1.16)	0.15	0.67 (0.39, 1.16)	0.15	0.64 (0.39, 1.06)	0.08	0.65 (0.39, 1.06)	0.08
≥ 7 days	0.67 (0.34, 1.33)	0.25	0.67 (0.34, 1.33)	0.25	0.79 (0.43, 1.44)	0.44	0.79 (0.44, 1.41)	0.42

*Note:* Model 1: crude model, age, sex, race, HR, SBP, DBP, MBP, CKD, LIV, HTN, AF, COPD, DM, CHF, HLP; RBC, WBC, platelet, hemoglobin, hematocrit, anion gap, bicarbonate. Model 2: model 1, BUN, calcium, chloride, creatinine, glucose, sodium, potassium, INR, PT, PTT, SOFA, SAPSII, OASIS, GCS, CRRT, ventilation, antiplatelet drug, anticoagulant drug, vasoactive drug. Abbreviations: CI, confidence interval; HR, hazard ratio.

### 3.6 Subgroup Analysis

**FIGURE 3:**
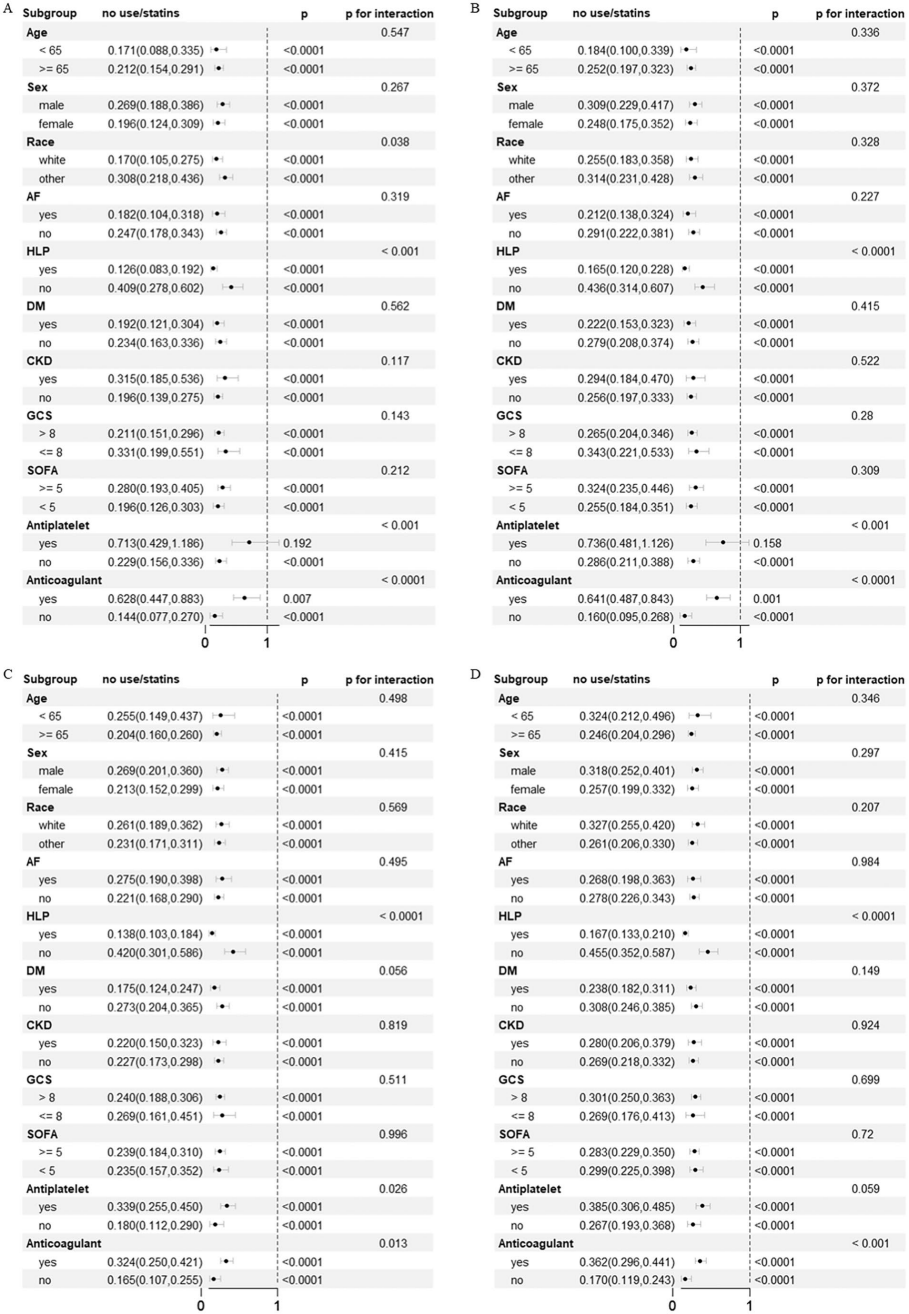
Association between statin use and baseline characteristics in patients with hemorrhagic stroke and ischemic stroke. (A) ICU mortality of hemorrhagic stroke. (B) In‐hospital mortality of hemorrhagic stroke. (C) ICU mortality of ischemic stroke. (D) In‐hospital mortality of ischemic stroke. AF, atrial fibrillation; CKD, chronic kidney disease; DM, diabetes mellitus; GCS, Glasgow Coma Scale; HLP, hyperlipidemia; OR, odds ratio; SOFA, sequential organ failure assessment.

We conducted a subgroup analysis to evaluate potential interactions between stratification variables and statin use (Figure 3). The findings demonstrated that the mortality risk reduction associated with statin therapy remained robust. In HS patients, statin use was significantly correlated with ICU and in‐hospital mortality among those with hyperlipidemia (*p* for interaction < 0.001), and a similar trend was observed in IS patients, suggesting that the protective effect of statins may be more pronounced in patients with hyperlipidemia. Additionally, it is noteworthy that in HS patients, concurrent use of antiplatelet or anticoagulant therapy attenuated the protective effect of statins (*p* for interaction < 0.001).

## 4 Discussion

This study provides compelling evidence regarding the impact of statin therapy on all‐cause mortality in critically ill patients with cerebrovascular disease. Given the high mortality and morbidity associated with both HS and IS, optimizing pharmacological interventions is crucial. While statins are primarily used for lipid‐lowering and cardiovascular protection, their pleiotropic effects, including anti‐inflammatory, neuroprotective, and endothelial‐stabilizing properties, suggest a potential role in improving outcomes for critically ill stroke patients. Our findings contribute to the growing body of literature supporting statin use in these patients and highlight the differential effects among stroke subtypes and statin types.

### 4.1 Mechanisms Underlying the Mortality Reduction With Statins

The observed reduction in 30‐day and 90‐day ICU and in‐hospital mortality among both HS and IS patients using statins can be attributed to pleiotropic properties. In HS patients, statins may mitigate secondary injury through blood–brain barrier (BBB) stabilization via RhoA/ROCK pathway inhibition [28], reducing perihematomal edema—a key determinant of neurological deterioration [20]. This aligns with preclinical evidence showing atorvastatin reduces matrix metalloproteinase‐9 (MMP‐9) activity, preserving BBB integrity after intracerebral hemorrhage [29]. Simultaneously, statins' anti‐inflammatory effects (e.g., IL‐6 and TNF‐α suppression) [30] may counterbalance the neurotoxic inflammation cascade triggered by hematoma breakdown products [31]. For IS patients, statins' benefits likely derive from dual mechanisms: acute neuroprotection through enhanced endothelial nitric oxide synthase (eNOS) activity [14], improving cerebral perfusion in the ischemic penumbra [32], and systemic effects stabilizing vulnerable atherosclerotic plaques [33]. Our finding of consistent mortality reduction across IS subgroups supports this dual‐action paradigm, where both central and peripheral mechanisms contribute to outcome improvement.

### 4.2 Differential Effects of Statin Types and Doses in HS and IS Patients

Our study revealed that atorvastatin and simvastatin significantly reduced mortality in HS patients, whereas rosuvastatin was associated with a mild but non‐significant increase in mortality risk. This disparity may be related to differences in lipophilicity and blood–brain barrier penetration [34]. Atorvastatin and simvastatin, being lipophilic statins, can cross the blood–brain barrier more readily, potentially exerting greater neuroprotective effects. This property may contribute to their neuroprotective effects in various neurological conditions [35]. Simvastatin has shown the most promising characteristics for preventing neurodegenerative conditions in vitro [36] and protecting blood–brain barrier integrity in experimental intracerebral hemorrhage [28]. Conversely, rosuvastatin, a hydrophilic statin, has limited central nervous system penetration and may not provide the same level of neuroprotection [37]. Additionally, some studies suggest that rosuvastatin may have a stronger anticoagulant effect by reducing thrombin generation [38], which could theoretically increase the risk of hematoma expansion in HS patients. However, this hypothesis requires further investigation. Furthermore, dose stratification analysis revealed a comparable magnitude of mortality benefit between standard‐dose and high‐dose statin therapy across both stroke subtypes. This finding suggests that standard doses might be sufficient to achieve optimal pharmacodynamic effects while minimizing dose‐related adverse events. Such observations resonate with recent evidence indicating that excessively lowering LDL‐cholesterol may lead to increased intracranial hemorrhage risk or worse outcomes [39]. Given the lack of additional survival benefit at higher doses observed in our study, clinicians might consider standard‐dose statin regimens to maximize therapeutic benefits and mitigate potential risks, especially in critically ill patients who may be more vulnerable to adverse drug reactions.

### 4.3 Timing of Statin Initiation and Mortality Risk in HS and IS Patients

A particularly striking finding of our study was that HS patients who were on statins before ICU admission had over a twofold increased risk of 30‐day and 90‐day ICU and in‐hospital mortality compared to those who initiated statins after ICU admission. Given that our analysis effectively controlled for potential confounders and baseline differences, the observed effect in HS patients is unlikely due to underlying comorbidities alone. A plausible explanation for this phenomenon is that early (i.e., pre‐ICU) statin use in the acute phase of HS may exacerbate bleeding risk. Statins, while beneficial for their anti‐inflammatory and endothelial‐protective properties, can also influence hemostatic balance. In the setting of HS, these effects might impair clot stabilization or promote hematoma expansion, thereby increasing mortality risk [40]. This suggests that for HS patients, initiating statin therapy in the acute phase may not confer additional benefit and might even be harmful, implying that a delayed initiation of statins after stabilization in the ICU could be more appropriate. Conversely, the absence of a significant association between the timing of statin initiation and outcomes in IS patients indicates that early administration remains beneficial in the ischemic context, likely due to its anti‐inflammatory, plaque‐stabilizing, and neuroprotective effects without the same risk of promoting hemorrhage [41]. Overall, these findings emphasize the importance of a tailored approach to statin therapy in cerebrovascular disease. In addition, we conducted sensitivity analyses stratified by the duration of statin use following ICU admission (≥ 3, ≥ 5, and ≥ 7 days). Our findings demonstrated that slightly extending the exposure threshold to ≥ 3 days effectively captured the pharmacodynamic benefits associated with statin therapy. Further extending exposure criteria to ≥ 5 or ≥ 7 days, however, did not confer additional measurable mortality benefits, and might introduce potential survival bias, given that patients surviving longer inherently possess a lower baseline mortality risk and thus receive prolonged statin treatment. Therefore, these sensitivity analyses strengthen causal inference, supporting the original selection of ≥ 2 days as the primary threshold. Clinically, shorter‐duration statin administration (approximately 3–5 days) appears to be an optimal therapeutic approach.

### 4.4 Subgroup Analysis

Based on our subgroup analyses, the survival benefit associated with statin therapy was notably pronounced among stroke patients with hyperlipidemia, suggesting that baseline lipid dysregulation may enhance responsiveness to statins. Mechanistically, this effect aligns with recent findings suggesting that optimal control of LDL cholesterol levels is critical in mediating outcomes post‐stroke. Specifically, studies have demonstrated a U‐shaped association between LDL cholesterol and all‐cause mortality, with excessively low LDL cholesterol potentially increasing risks due to heightened vulnerability to infections [42]. Conversely, appropriately controlled LDL cholesterol—often achieved in patients with hyperlipidemia undergoing statin therapy—has been linked to reduced infection rates and improved stroke outcomes [43]. Moreover, recent evidence suggests that aggressively lowering LDL cholesterol may paradoxically elevate intracranial hemorrhage risks, emphasizing the necessity for personalized statin treatment strategies [39]. Collectively, these pivotal studies underscore the nuanced role lipid metabolism and statin therapy play in stroke recovery, particularly among hyperlipidemic patients.

## 5 Strengths and Limitations

This study possesses several notable strengths, including the utilization of a large, high‐quality critical care database (MIMIC‐IV), the employment of rigorous statistical methodologies to adjust for potential confounders, and the comprehensive evaluation of statin effects in both HS and IS populations. Nonetheless, several limitations merit consideration. First, as a retrospective observational study, the possibility of residual confounding cannot be completely eliminated despite robust statistical adjustments, including propensity score matching and multivariable analyses. Second, our findings are specific to critically ill patients admitted to the ICU, and thus may not be fully generalizable to the broader population of stroke patients outside intensive care settings. Third, the absence of granular data on statin adherence, dose titration, and potential drug–drug interactions may have influenced the observed associations. Furthermore, although LDL‐C is a well‐recognized determinant of vascular risk and stroke outcomes, our ability to account for LDL‐C was limited due to a high rate of missing serum lipid measurements in the MIMIC‐IV cohort. Future prospective studies and randomized controlled trials with comprehensive clinical and laboratory data are warranted to validate our findings and to inform evidence‐based guidelines for statin use in acute stroke care.

## 6 Conclusion

Our study provides robust evidence supporting the beneficial effects of statin therapy in critically ill patients with cerebrovascular disease. However, the differential effects observed among statin types, doses, and timing of initiation highlight the need for personalized treatment approaches. Future prospective studies and randomized controlled trials are warranted to further elucidate the optimal statin regimen in this high‐risk population.

## Author Contributions

D.T.: conceptualization, methodology, formal analysis, investigation, supervision, writing – original draft, writing – review and editing. Z.H.: conceptualization, formal analysis, investigation, writing – original draft. S.L.: formal analysis, investigation. F.C.: conceptualization, writing – review and editing. All authors have read and agreed to the published version of the manuscript.

## Ethics Statement

The MIMIC‐IV project received approval from the Institutional Review Boards of both the Massachusetts Institute of Technology and Beth Israel Deaconess Medical Center. Patient information was anonymized, thereby obviating the need for informed consent from individual patients for this study.

## Consent

The authors have nothing to report.

## Conflicts of Interest

The authors declare no conflicts of interest.

## Supporting information


**Figure S1:** cns70542‐sup‐0001‐DataS1.docx.

**Data S2:** cns70542‐sup‐0002‐DataS2.docx.

**Table S1–S5:** cns70542‐sup‐0003‐DataS3.docx.

## Data Availability

The data that support the findings of this study are available from the corresponding author upon reasonable request.
