# Supplementary material for: Assessment of the Impact of Statin Use to Predict All‐Cause Mortality in Patients With Critical Cerebrovascular Disease: A Retrospective Cohort Study From the MIMIC‐IV Database
Source: CNS Neurosci Ther. 2025 Jul 27;31(7):e70542. doi: 10.1111/cns.70542 (PMC12301503; doi:10.1111/cns.70542)
Supplement: Supplementary file 1 — Figure S1: cns70542‐sup‐0001‐DataS1.docx. [file CNS-31-e70542-s002.docx]

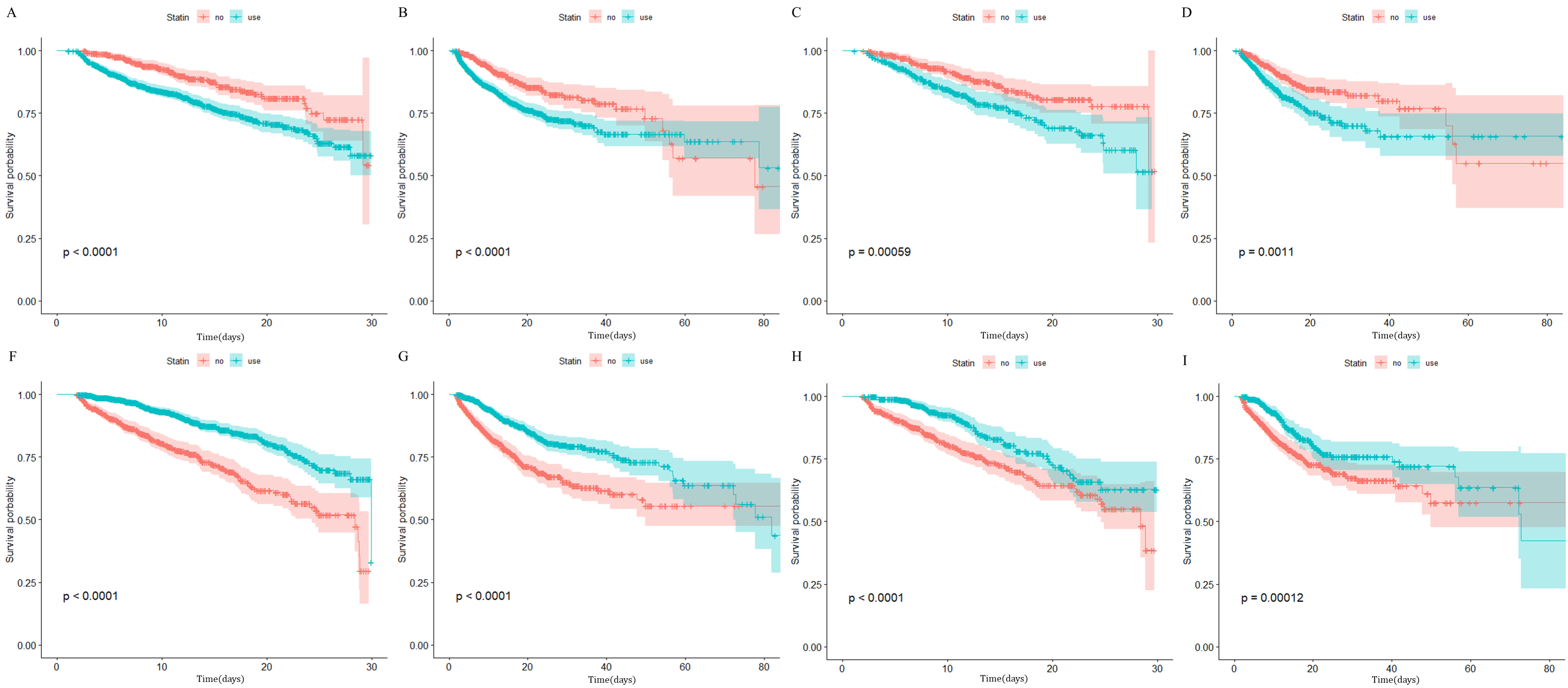


Figure S1. Kaplan-Meier survival curves of the non-statin group and statin group for hospital mortality on Landmark analyses. (A) 30-day in-hospital mortality of hemorrhagic stroke; (B) 90-day in-hospital mortality of hemorrhagic stroke; (C) 30-day in-hospital mortality of hemorrhagic stroke after PSM; (D) 30-day in-hospital mortality of hemorrhagic stroke after PSM; (E) 30-day in-hospital mortality of ischemic stroke; (F) 90-day in-hospital mortality of ischemic stroke; (G) 30-day in-hospital mortality of ischemic stroke after PSM; (H) 30-day in-hospital mortality of ischemic stroke after PSM.
