# Supplementary material for: Assessment of the Impact of Statin Use to Predict All‐Cause Mortality in Patients With Critical Cerebrovascular Disease: A Retrospective Cohort Study From the MIMIC‐IV Database
Source: CNS Neurosci Ther. 2025 Jul 27;31(7):e70542. doi: 10.1111/cns.70542 (PMC12301503; doi:10.1111/cns.70542)
Supplement: Supplementary file 2 — Data S2: cns70542‐sup‐0002‐DataS2.docx. [file CNS-31-e70542-s003.docx]

**Supplementary materials 1. ICD-9 and ICD-10 code for hemorrhagic stroke and ischemic stroke.**

|  | **Hemorrhagic stroke** |
| --- | --- |
| **ICD-9** |  |
| 430 | Subarachnoid hemorrhage |
| 431 | Intracerebral hemorrhage |
| 4320 | Nontraumatic extradural hemorrhage |
| 4321 | Subdural hemorrhage |
| 4329 | Unspecified intracranial hemorrhage |
| **ICD-10** |  |
| I610 | Nontraumatic intracerebral hemorrhage in hemisphere, subcortical |
| I611 | Nontraumatic intracerebral hemorrhage in hemisphere, cortical |
| I612 | Nontraumatic intracerebral hemorrhage in hemisphere, unspecified |
| I613 | Nontraumatic intracerebral hemorrhage in brain stem |
| I614 | Nontraumatic intracerebral hemorrhage in cerebellum |
| I615 | Nontraumatic intracerebral hemorrhage, intraventricular |
| I616 | Nontraumatic intracerebral hemorrhage, multiple localized |
| I617 | Other nontraumatic intracerebral hemorrhage |
| I618 | Nontraumatic intracerebral hemorrhage, unspecified |
| I619 | Nontraumatic intracerebral hemorrhage in hemisphere, subcortical |
| I60 | Nontraumatic subarachnoid hemorrhage |
| I600 | Nontraumatic subarachnoid hemorrhage from carotid siphon and bifurcation |
| I6000 | Nontraumatic subarachnoid hemorrhage from unspecified carotid siphon and bifurcation |
| I6001 | Nontraumatic subarachnoid hemorrhage from right carotid siphon and bifurcation |
| I6002 | Nontraumatic subarachnoid hemorrhage from left carotid siphon and bifurcation |
| I601 | Nontraumatic subarachnoid hemorrhage from middle cerebral artery |
| I6010 | Nontraumatic subarachnoid hemorrhage from unspecified middle cerebral artery |
| I6011 | Nontraumatic subarachnoid hemorrhage from right middle cerebral artery |
| I6012 | Nontraumatic subarachnoid hemorrhage from left middle cerebral artery |
| I602 | Nontraumatic subarachnoid hemorrhage from anterior communicating artery |
| I6020 | Nontraumatic subarachnoid hemorrhage from unspecified anterior communicating artery |
| I6021 | Nontraumatic subarachnoid hemorrhage from right anterior communicating artery |
| I6022 | Nontraumatic subarachnoid hemorrhage from left anterior communicating artery |
| I603 | Nontraumatic subarachnoid hemorrhage from posterior communicating artery |
| I6030 | Nontraumatic subarachnoid hemorrhage from unspecified posterior communicating artery |
| I6031 | Nontraumatic subarachnoid hemorrhage from right posterior communicating artery |
| I6032 | Nontraumatic subarachnoid hemorrhage from left posterior communicating artery |
| I604 | Nontraumatic subarachnoid hemorrhage from basilar artery |
| I605 | Nontraumatic subarachnoid hemorrhage from vertebral artery |
| I6050 | Nontraumatic subarachnoid hemorrhage from unspecified vertebral artery |
| I6051 | Nontraumatic subarachnoid hemorrhage from right vertebral artery |
| I6052 | Nontraumatic subarachnoid hemorrhage from left vertebral artery |
| I606 | Nontraumatic subarachnoid hemorrhage from other intracranial arteries |
| I607 | Nontraumatic subarachnoid hemorrhage from unspecified intracranial artery |
| I608 | Other nontraumatic subarachnoid hemorrhage |
| I609 | Nontraumatic subarachnoid hemorrhage, unspecified |
| I61 | Nontraumatic intracerebral hemorrhage |
| I610 | Nontraumatic intracerebral hemorrhage in hemisphere, subcortical |
| I611 | Nontraumatic intracerebral hemorrhage in hemisphere, cortical |
| I612 | Nontraumatic intracerebral hemorrhage in hemisphere, unspecified |
| I613 | Nontraumatic intracerebral hemorrhage in brain stem |
| I614 | Nontraumatic intracerebral hemorrhage in cerebellum |
| I615 | Nontraumatic intracerebral hemorrhage, intraventricular |
| I616 | Nontraumatic intracerebral hemorrhage, multiple localized |
| I618 | Other nontraumatic intracerebral hemorrhage |
| I619 | Nontraumatic intracerebral hemorrhage, unspecified |
| I62 | Other and unspecified nontraumatic intracranial hemorrhage |
| I620 | Nontraumatic subdural hemorrhage |
| I6200 | Nontraumatic subdural hemorrhage, unspecified |
| I6201 | Nontraumatic acute subdural hemorrhage |
| I6202 | Nontraumatic subacute subdural hemorrhage |
| I6203 | Nontraumatic chronic subdural hemorrhage |
| I621 | Nontraumatic extradural hemorrhage |
| I629 | Nontraumatic intracranial hemorrhage, unspecified |
|  | **Ischemic stroke** |
| **ICD-9** |  |
| 9-43311 | Occlusion and stenosis of carotid artery with cerebral infarction |
| 9-43401 | Cerebral thrombosis with cerebral infarction |
| 9-43331 | Occlusion and stenosis of multiple and bilateral precerebral arteries with cerebral infarction |
| 9-43321 | Occlusion and stenosis of vertebral artery with cerebral infarction |
| 9-43381 | Occlusion and stenosis of other specified precerebral artery with cerebral infarction |
| 9-34670 | Chronic migraine without aura, without mention of intractable migraine without mention of status migrainosus |
| 9-43391 | Occlusion and stenosis of unspecified precerebral artery with cerebral infarction |
| 9-4358 | Other specified transient cerebral ischemias |
| **ICD-10** |  |
| I63011 | Cerebral infarction due to thrombosis of right vertebral artery |
| I63012 | Cerebral infarction due to thrombosis of left vertebral artery |
| I63013 | Cerebral infarction due to thrombosis of bilateral vertebral arteries |
| I6302 | Cerebral infarction due to thrombosis of basilar artery |
| I63031 | Cerebral infarction due to thrombosis of right carotid artery |
| I63039 | Cerebral infarction due to thrombosis of unspecified carotid artery |
| I6309 | Cerebral infarction due to thrombosis of other precerebral artery |
| I6310 | Cerebral infarction due to embolism of unspecified precerebral artery |
| I63111 | Cerebral infarction due to embolism of right vertebral artery |
| I63112 | Cerebral infarction due to embolism of left vertebral artery |
| I63113 | Cerebral infarction due to embolism of bilateral vertebral arteries |
| I63119 | Cerebral infarction due to embolism of unspecified vertebral artery |
| I6312 | Cerebral infarction due to embolism of basilar artery |
| I63132 | Cerebral infarction due to embolism of left carotid artery |
| I63133 | Cerebral infarction due to embolism of bilateral carotid arteries |
| I63139 | Cerebral infarction due to embolism of unspecified carotid artery |
| I6319 | Cerebral infarction due to embolism of other precerebral artery |
| I6320 | Cerebral infarction due to unspecified occlusion or stenosis of unspecified precerebral arteries |
| I63211 | Cerebral infarction due to unspecified occlusion or stenosis of right vertebral artery |
| I63212 | Cerebral infarction due to unspecified occlusion or stenosis of left vertebral artery |
| I63213 | Cerebral infarction due to unspecified occlusion or stenosis of bilateral vertebral arteries |
| I63219 | Cerebral infarction due to unspecified occlusion or stenosis of unspecified vertebral artery |
| I6322 | Cerebral infarction due to unspecified occlusion or stenosis of basilar artery |
| I63231 | Cerebral infarction due to unspecified occlusion or stenosis of right carotid arteries |
| I63232 | Cerebral infarction due to unspecified occlusion or stenosis of left carotid arteries |
| I63233 | Cerebral infarction due to unspecified occlusion or stenosis of bilateral carotid arteries |
| I63239 | Cerebral infarction due to unspecified occlusion or stenosis of unspecified carotid artery |
| I6329 | Cerebral infarction due to unspecified occlusion or stenosis of other precerebral arteries |
| I6330 | Cerebral infarction due to thrombosis of unspecified cerebral artery |
| I63311 | Cerebral infarction due to thrombosis of right middle cerebral artery |
| I63312 | Cerebral infarction due to thrombosis of left middle cerebral artery |
| I63313 | Cerebral infarction due to thrombosis of bilateral middle cerebral arteries |
| I63319 | Cerebral infarction due to thrombosis of unspecified middle cerebral artery |
| I63321 | Cerebral infarction due to thrombosis of right anterior cerebral artery |
| I63322 | Cerebral infarction due to thrombosis of left anterior cerebral artery |
| I63331 | Cerebral infarction due to thrombosis of right posterior cerebral artery |
| I63332 | Cerebral infarction due to thrombosis of left posterior cerebral artery |
| I63333 | Cerebral infarction due to thrombosis of bilateral posterior cerebral arteries |
| I63341 | Cerebral infarction due to thrombosis of right cerebellar artery |
| I63342 | Cerebral infarction due to thrombosis of left cerebellar artery |
| I63343 | Cerebral infarction due to thrombosis of bilateral cerebellar arteries |
| I63349 | Cerebral infarction due to thrombosis of unspecified cerebellar artery |
| I6339 | Cerebral infarction due to thrombosis of other cerebral artery |
| I6340 | Cerebral infarction due to embolism of unspecified cerebral artery |
| I63411 | Cerebral infarction due to embolism of right middle cerebral artery |
| I63413 | Cerebral infarction due to embolism of bilateral middle cerebral arteries |
| I63419 | Cerebral infarction due to embolism of unspecified middle cerebral artery |
| I63421 | Cerebral infarction due to embolism of right anterior cerebral artery |
| I63422 | Cerebral infarction due to embolism of left anterior cerebral artery |
| I63423 | Cerebral infarction due to embolism of bilateral anterior cerebral arteries |
| I63429 | Cerebral infarction due to embolism of unspecified anterior cerebral artery |
| I63431 | Cerebral infarction due to embolism of right posterior cerebral artery |
| I63432 | Cerebral infarction due to embolism of left posterior cerebral artery |
| I63433 | Cerebral infarction due to embolism of bilateral posterior cerebral arteries |
| I63439 | Cerebral infarction due to embolism of unspecified posterior cerebral artery |
| I63442 | Cerebral infarction due to embolism of left cerebellar artery |
| I63443 | Cerebral infarction due to embolism of bilateral cerebellar arteries |
| I63449 | Cerebral infarction due to embolism of unspecified cerebellar artery |
| I6349 | Cerebral infarction due to embolism of other cerebral artery |
| I6350 | Cerebral infarction due to unspecified occlusion or stenosis of unspecified cerebral artery |
| I63511 | Cerebral infarction due to unspecified occlusion or stenosis of right middle cerebral artery |
| I63513 | Cerebral infarction due to unspecified occlusion or stenosis of bilateral middle cerebral arteries |
| I63521 | Cerebral infarction due to unspecified occlusion or stenosis of right anterior cerebral artery |
| I63522 | Cerebral infarction due to unspecified occlusion or stenosis of left anterior cerebral artery |
| I63523 | Cerebral infarction due to unspecified occlusion or stenosis of bilateral anterior cerebral arteries |
| I63529 | Cerebral infarction due to unspecified occlusion or stenosis of unspecified anterior cerebral artery |
| I63531 | Cerebral infarction due to unspecified occlusion or stenosis of right posterior cerebral artery |
| I63532 | Cerebral infarction due to unspecified occlusion or stenosis of left posterior cerebral artery |
| I63533 | Cerebral infarction due to unspecified occlusion or stenosis of bilateral posterior cerebral arteries |
| I63539 | Cerebral infarction due to unspecified occlusion or stenosis of unspecified posterior cerebral artery |
| I63541 | Cerebral infarction due to unspecified occlusion or stenosis of right cerebellar artery |
| I63542 | Cerebral infarction due to unspecified occlusion or stenosis of left cerebellar artery |
| I63543 | Cerebral infarction due to unspecified occlusion or stenosis of bilateral cerebellar arteries |
| I63549 | Cerebral infarction due to unspecified occlusion or stenosis of unspecified cerebellar artery |
| I6359 | Cerebral infarction due to unspecified occlusion or stenosis of other cerebral artery |
| I636 | Cerebral infarction due to cerebral venous thrombosis, nonpyogenic |
| I638 | Other cerebral infarction |
| I6389 | Other cerebral infarction |
| I639 | Cerebral infarction, unspecified |
| I659 | Occlusion and stenosis of unspecified precerebral artery |
| I69331 | Monoplegia of upper limb following cerebral infarction affecting right dominant side |
| I69351 | Hemiplegia and hemiparesis following cerebral infarction affecting right dominant side |
| I69354 | Hemiplegia and hemiparesis following cerebral infarction affecting left non-dominant side |
| Z8673 | Personal history of transient ischemic attack (TIA), and cerebral infarction without residual deficits |
