# Supplementary material for: Assessment of the Impact of Statin Use to Predict All‐Cause Mortality in Patients With Critical Cerebrovascular Disease: A Retrospective Cohort Study From the MIMIC‐IV Database
Source: CNS Neurosci Ther. 2025 Jul 27;31(7):e70542. doi: 10.1111/cns.70542 (PMC12301503; doi:10.1111/cns.70542)
Supplement: Supplementary file 3 — Table S1–S5: cns70542‐sup‐0003‐DataS3.docx. [file CNS-31-e70542-s001.docx]

**Table S1. Baseline characteristics of patients with hemorrhagic stroke and ischemic stroke on** **Landmark analyses after PSM.**

| **Variables** | **Hemorrhagic Stroke** | | | | **Ischemic Stroke** | | | |
| --- | --- | --- | --- | --- | --- | --- | --- | --- |
|  | Total  (n =956) | No use  (n = 478) | Statin use  (n = 478) | P | Total  (n = 1186) | No use  (n = 593) | Statin use  (n = 593) | **P** |
| Demographics |  |  |  |  |  |  |  |  |
| Age, years | 69.97 ± 13.97 | 70.35 ± 14.68 | 69.59 ± 13.23 | 0.40 | 70.66 ± 14.84 | 70.33 ± 16.35 | 71.00 ± 13.16 | 0.44 |
| Sex, female, n (%) | 492(51.46) | 237(49.58) | 255(53.35) | 0.27 | 609(51.35) | 294(49.58) | 315(53.12) | 0.25 |
| Race |  |  |  | 1.00 |  |  |  | 1.00 |
| other, n (%) | 397(41.53) | 198(41.42) | 199(41.63) |  | 614(51.77) | 307(51.77) | 307(51.77) |  |
| white, n (%) | 559(58.47) | 280(58.58) | 279(58.37) |  | 572(48.23) | 286(48.23) | 286(48.23) |  |
| Vital signs |  |  |  |  |  |  |  |  |
| HR mean, beats/min | 79.00 ± 13.17 | 78.87 ± 13.39 | 79.12 ± 12.97 | 0.76 | 82.38 ± 15.33 | 82.71 ± 15.95 | 82.05 ± 14.68 | 0.46 |
| SBP mean, mmHg | 130.37 ± 13.51 | 130.40 ± 13.08 | 130.34 ± 13.95 | 0.94 | 124.72 ± 17.49 | 124.57 ± 16.83 | 124.86 ± 18.14 | 0.78 |
| DBP mean, mmHg | 67.29 ± 11.38 | 67.15 ± 11.11 | 67.42 ± 11.66 | 0.72 | 66.88 ± 12.63 | 66.68 ± 12.32 | 67.07 ± 12.93 | 0.59 |
| MBP mean, mmHg | 85.45 ± 10.32 | 85.36 ± 10.00 | 85.54 ± 10.63 | 0.78 | 83.90 ± 12.15 | 83.69 ± 11.69 | 84.11 ± 12.60 | 0.56 |
| Comorbidity |  |  |  |  |  |  |  |  |
| CKD, n (%) | 115(12.03) | 60(12.55) | 55(11.51) | 0.69 | 260(21.92) | 114(19.22) | 146(24.62) | 0.03 |
| LD, n (%) | 8(0.84) | 5(1.05) | 3(0.63) | 0.72 | 12(1.01) | 9(1.52) | 3(0.51) | 0.15 |
| HTN, n (%) | 595(62.24) | 295(61.72) | 300(62.76) | 0.79 | 554(46.71) | 276(46.54) | 278(46.88) | 0.95 |
| AF, n (%) | 206(21.55) | 104(21.76) | 102(21.34) | 0.94 | 250(21.08) | 128(21.59) | 122(20.57) | 0.72 |
| COPD, n (%) | 47(4.92) | 24(5.02) | 23(4.81) | 1.00 | 135(11.38) | 64(10.79) | 71(11.97) | 0.58 |
| DM, n (%) | 287(30.02) | 140(29.29) | 147(30.75) | 0.67 | 423(35.67) | 192(32.38) | 231(38.95) | 0.02 |
| CHF, n (%) | 148(15.48) | 74(15.48) | 74(15.48) | 1.00 | 343(28.92) | 156(26.31) | 187(31.53) | 0.05 |
| HLP, n (%) | 557(58.26) | 274(57.32) | 283(59.21) | 0.60 | 562(47.39) | 263(44.35) | 299(50.42) | 0.04 |
| Laboratory indicators |  |  |  |  |  |  |  |  |
| RBC (10^12/L) | 4.02 ± 0.60 | 4.01 ± 0.59 | 4.02 ± 0.60 | 0.98 | 3.74 ± 0.69 | 3.75 ± 0.70 | 3.73 ± 0.68 | 0.62 |
| WBC (10^9/L) | 12.03 ± 7.79 | 12.11 ± 9.88 | 11.96 ± 4.90 | 0.77 | 12.80 ± 6.15 | 12.75 ± 6.25 | 12.86 ± 6.04 | 0.75 |
| Platelet (10^9/L) | 214.01 ± 70.00 | 211.67 ± 69.88 | 216.35 ± 70.12 | 0.30 | 210.14 ± 86.07 | 210.86 ± 88.14 | 209.42 ± 84.02 | 0.77 |
| Hemoglobin (g/L) | 12.09 ± 1.69 | 12.13 ± 1.63 | 12.05 ± 1.74 | 0.44 | 11.05 ± 2.01 | 11.08 ± 2.03 | 11.01 ± 1.98 | 0.57 |
| Hematocrit (%) | 39.15 ± 5.58 | 39.28 ± 5.50 | 39.01 ± 5.67 | 0.46 | 36.72 ± 6.39 | 36.85 ± 6.34 | 36.59 ± 6.44 | 0.49 |
| Aniongap (mmol/L) | 15.70 ± 4.10 | 15.77 ± 3.94 | 15.63 ± 4.26 | 0.58 | 16.25 ± 4.88 | 16.13 ± 4.56 | 16.36 ± 5.19 | 0.42 |
| Bicarbonate (mmol/L) | 24.21 ± 3.13 | 24.19 ± 2.90 | 24.22 ± 3.34 | 0.92 | 23.33 ± 3.36 | 23.28 ± 3.37 | 23.38 ± 3.35 | 0.61 |
| Bun (mg/dl) | 21.02 ± 15.30 | 21.22 ± 14.80 | 20.82 ± 15.79 | 0.68 | 27.65 ± 22.68 | 27.18 ± 22.74 | 28.11 ± 22.63 | 0.48 |
| Calcium (mg/dL) | 8.91 ± 0.63 | 8.93 ± 0.62 | 8.89 ± 0.64 | 0.34 | 8.79 ± 0.69 | 8.76 ± 0.70 | 8.82 ± 0.67 | 0.14 |
| Chloride (mmol/L) | 106.15 ± 5.81 | 106.21 ± 5.73 | 106.09 ± 5.89 | 0.74 | 105.69 ± 6.19 | 105.72 ± 6.33 | 105.66 ± 6.05 | 0.88 |
| Creatinine (mg/dL) | 1.18 ± 1.24 | 1.18 ± 1.20 | 1.17 ± 1.27 | 0.90 | 1.47 ± 1.40 | 1.43 ± 1.45 | 1.52 ± 1.34 | 0.29 |
| Glucose (mmol/L) | 168.59 ± 95.46 | 167.06 ± 110.92 | 170.11 ± 77.04 | 0.62 | 175.03 ± 98.43 | 169.02 ± 77.36 | 181.04 ± 115.49 | 0.04 |
| Sodium (mmol/L) | 141.35 ± 5.05 | 141.44 ± 4.83 | 141.26 ± 5.26 | 0.58 | 140.67 ± 5.39 | 140.64 ± 5.41 | 140.70 ± 5.38 | 0.87 |
| Potassium (mmol/L) | 4.31 ± 0.75 | 4.29 ± 0.72 | 4.32 ± 0.77 | 0.60 | 4.61 ± 0.87 | 4.56 ± 0.80 | 4.66 ± 0.94 | 0.03 |
| INR | 1.28 ± 0.42 | 1.27 ± 0.41 | 1.28 ± 0.42 | 0.75 | 1.49 ± 0.79 | 1.49 ± 0.82 | 1.49 ± 0.75 | 0.94 |
| PT (seconds) | 13.96 ± 4.24 | 13.92 ± 4.25 | 14.00 ± 4.24 | 0.75 | 16.30 ± 8.63 | 16.28 ± 9.08 | 16.31 ± 8.17 | 0.96 |
| PTT (seconds) | 33.22 ± 16.96 | 33.33 ± 17.50 | 33.11 ± 16.42 | 0.85 | 44.06 ± 31.01 | 41.15 ± 28.22 | 46.96 ± 33.33 | <0.01 |
| Disease severity score |  |  |  |  |  |  |  |  |
| SOFA | 3.37 ± 2.52 | 3.37 ± 2.55 | 3.37 ± 2.49 | 1.00 | 4.72 ± 3.42 | 4.69 ± 3.53 | 4.75 ± 3.31 | 0.77 |
| SAPSII | 33.91 ± 11.67 | 34.06 ± 11.10 | 33.76 ± 12.22 | 0.69 | 37.45 ± 13.06 | 37.43 ± 13.61 | 37.47 ± 12.50 | 0.95 |
| OASIS | 32.08 ± 7.86 | 32.24 ± 7.71 | 31.93 ± 8.01 | 0.54 | 33.26 ± 8.30 | 33.28 ± 8.16 | 33.25 ± 8.45 | 0.95 |
| GCS | 12.41 ± 3.16 | 12.42 ± 3.08 | 12.40 ± 3.24 | 0.92 | 13.09 ± 2.93 | 13.01 ± 3.06 | 13.17 ± 2.80 | 0.35 |
| Treatment |  |  |  |  |  |  |  |  |
| CRRT, n (%) | 11(1.15) | 7(1.46) | 4(0.84) | 0.54 | 39(3.29) | 20(3.37) | 19(3.20) | 1.00 |
| Ventilation, n (%) | 407(42.57) | 202(42.26) | 205(42.89) | 0.90 | 499(42.07) | 251(42.33) | 248(41.82) | 0.91 |
| Antiplatelet drug, n (%) | 407(42.57) | 192(40.17) | 215(44.98) | 0.15 | 763(64.33) | 371(62.56) | 392(66.10) | 0.23 |
| Anticoagulant drug, n (%) | 864(90.38) | 428(89.54) | 436(91.21) | 0.44 | 1019(85.92) | 510(86.00) | 509(85.83) | 1.00 |
| Vasoactive drug, n (%) | 213(22.28) | 93(19.46) | 120(25.10) | 0.04 | 386(32.55) | 188(31.70) | 198(33.39) | 0.58 |
| Length of stay (LOS) |  |  |  |  |  |  |  |  |
| LOS in hospital | 15.96 ± 14.67 | 15.59 ± 15.92 | 16.34 ± 13.31 | 0.43 | 15.39 ± 14.93 | 15.36 ± 15.07 | 15.43 ± 14.79 | 0.93 |
| LOS in ICU | 9.18 ± 7.74 | 8.98 ± 7.30 | 9.38 ± 8.15 | 0.43 | 7.41 ± 7.15 | 7.65 ± 7.15 | 7.16 ± 7.15 | 0.24 |
| Outcomes |  |  |  |  |  |  |  |  |
| ICU death, n (%) | 93(9.73) | 59(12.34) | 34(7.11) | <0.01 | 132(11.13) | 43(7.25) | 89(15.01) | <0.0001 |
| In-hospital death, n (%) | 145(15.17) | 89(18.62) | 56(11.72) | <0.01 | 200(16.86) | 75(12.65) | 125(21.08) | <0.001 |
| 30-day mortality, n (%) | 96(10.04) | 62(12.97) | 34(7.11) | <0.01 | 140(11.80) | 94(15.85) | 46(7.76) | <0.0001 |
| 90-day mortality, n (%) | 98(10.25) | 63(13.18) | 35(7.32) | <0.01 | 141(11.89) | 95(16.02) | 46(7.76) | <0.0001 |

HR, heart rate; SBP, systolic blood pressure; DBP, diastolic blood pressure; MBP, mean blood pressure; CKD, chronic kidney disease; LD, liver disease; HTN, hypertension; AF, atrial fibrillation; COPD, chronic obstructive pulmonary disease; DM, diabetes mellitus; CHF, congestive heart failure; HLP, hyperlipidemia; INR, international normalized ratio; PT, prothrombin time; PTT, partial thromboplastin time. SOFA, sequential organ failure assessment; SAPSII, simplified acute physiology score; OASIS, oxford acute severity of illness score; GCS, Glasgow coma scale; CRRT, continuous renal replacement therapy.

**Table S2. Association between the statin group and all-cause hospital mortality on Landmark analyses.**

| **Mortality** |  | **HS-30-day mortality** | | **HS-90-day mortality** | | **IS-30-day mortality** | | **IS-90-day mortality** | |
| --- | --- | --- | --- | --- | --- | --- | --- | --- | --- |
|  | No use | HR 95%CI | P | HR 95%CI | P | HR 95%CI | P | HR 95%CI | P |
| **CM** | Ref | 0.51(0.39,0.67) | <0.0001 | 0.55(0.43,0.72) | <0.0001 | 0.43(0.35,0.54) | <0.0001 | 0.47(0.38,0.58) | <0.0001 |
| **Model 1** | Ref | 0.42(0.32,0.56) | <0.0001 | 0.46(0.35,0.60) | <0.0001 | 0.37(0.29,0.46) | <0.0001 | 0.39(0.32,0.49) | <0.0001 |
| **Model 2** | Ref | 0.44(0.34,0.59) | <0.0001 | 0.48(0.37,0.63) | <0.0001 | 0.38(0.30,0.48) | <0.0001 | 0.41(0.33,0.51) | <0.0001 |
| **Model 3** | Ref | 0.40(0.29,0.54) | <0.0001 | 0.43(0.32,0.57) | <0.0001 | 0.35(0.27,0.44) | <0.0001 | 0.37(0.30,0.47) | <0.0001 |
| **Model 4** | Ref | 0.41(0.30,0.55) | <0.0001 | 0.44(0.33,0.59) | <0.0001 | 0.34(0.27,0.43) | <0.0001 | 0.37(0.29,0.46) | <0.0001 |
| **Model 5** | Ref | 0.41(0.30,0.56) | <0.0001 | 0.44(0.33,0.60) | <0.0001 | 0.34(0.27,0.44) | <0.0001 | 0.36(0.29,0.46) | <0.0001 |
| **Model 6** | Ref | 0.60(0.44,0.83) | 0.002 | 0.64(0.47,0.87) | 0.004 | 0.41(0.31,0.53) | <0.0001 | 0.44(0.34,0.56) | <0.0001 |
| **PSM** | Ref | 0.57(0.39, 0.83) | 0.004 | 0.61(0.42, 0.88) | 0.010 | 0.45(0.33, 0.62) | < 0.001 | 0.49(0.36, 0.66) | <0.0001 |
| **Fine-Gray** | Ref | 0.55(0.38, 0.79) | 0.001 | 0.54(0.38, 0.79) | 0.001 | 0.44(0.31, 0.65) | < 0.0001 | 0.44(0.30, 0.64) | <0.0001 |

HS, Hemorrhagic Stroke; IS, Ischemic Stroke; CM, crude model; HR, hazard ratio; CI, confidence interval; Crudel model: No adjusted; Model 1: Crudel model, Age, Sex, Race; Model 2: model 1, HR, SBP, DBP, MBP; Model 3: model 2, CKD, LD, HTN, AF, COPD, DM, CHF, HLP; Model 4: model 3, RBC, WBC, Platelet, Hemoglobin, Hematocrit, Aniongap, Bicarbonate, Bun, Calcium, Chloride, Creatinine, Glucose, Sodium, Potassium, INR, PT, PTT; Model 5: model 4, SOFA, SAPSII, OASIS, GCS; Model 6: model 5, CRRT, Ventilation, Antiplatelet drug, Anticoagulant drug, Vasoactive drug;

**Table S3. The association between different statins and all-cause hospital mortality.**

| **Mortality** | **HS-30-day mortality** | | **HS-90-day mortality** | | **IS-30-day mortality** | | **IS-90-day mortality** | |
| --- | --- | --- | --- | --- | --- | --- | --- | --- |
|  | HR 95%CI | P | HR 95%CI | P | HR 95%CI | P | HR 95%CI | P |
| Non-statin | Ref | |  |  |  |  |  |  |
| **Types of statins** | |  |  |  |  |  |  |  |
| Atorvastatin | 0.54(0.38,0.79) | 0.001 | 0.54(0.38,0.78) | 0.001 | 0.33(0.26,0.43) | <0.0001 | 0.35(0.27,0.45) | <0.0001 |
| Pravastatin | 0.58(0.28,1.20) | 0.14 | 0.63(0.32,1.25) | 0.19 | 0.23(0.10,0.53) | <0.001 | 0.25(0.12,0.54) | <0.001 |
| Rosuvastatin Calcium | 0.71(0.29,1.76) | 0.46 | 0.82(0.35,1.88) | 0.63 | 0.41(0.22,0.73) | 0.003 | 0.39(0.22,0.69) | 0.001 |
| Simvastatin | 0.4(0.24,0.67) | <0.001 | 0.41(0.25,0.68) | <0.001 | 0.31(0.18,0.56) | <0.0001 | 0.31(0.17,0.55) | <0.0001 |
| **Doses of statins** | |  |  |  |  |  |  |  |
| High Dose | 0.53(0.36,0.77) | 0.001 | 0.54(0.37,0.79) | 0.001 | 0.32(0.24,0.42) | <0.0001 | 0.33(0.26,0.43) | <0.0001 |
| Standard dose | 0.5(0.34,0.72) | <0.001 | 0.51(0.35,0.73) | <0.001 | 0.35(0.25,0.51) | <0.0001 | 0.36(0.25,0.52) | <0.0001 |

**Table S4. The association between pre-ICU statin and post-ICU statin for all-cause hospital mortality.**

| **Variable** | **HS-30-day mortality** | | **HS-90-day mortality** | | **IS-30-day mortality** | | **IS-90-day mortality** | |
| --- | --- | --- | --- | --- | --- | --- | --- | --- |
|  | HR 95%CI | P | HR 95%CI | P | HR 95%CI | P | HR 95%CI | P |
| **Model1** |  |  |  |  |  |  |  |  |
| post-icu statin | Ref |  | Ref |  | Ref |  | Ref |  |
| pre-icu statin | 2.62(1.41, 4.89) | <0.01 | 2.85(1.59,5.12) | <0.01 | 1.00(0.71,1.40) | 0.98 | 1.00(0.70,1.42) | 0.98 |
| **Model2** |  |  |  |  |  |  |  |  |
| post-icu statin | Ref |  | Ref |  | Ref |  | Ref |  |
| pre-icu statin | 2.74(1.44, 5.19) | <0.01 | 3.72(1.86, 7.46) | <0.01 | 0.99(0.71,1.36) | 0.94 | 0.95(0.68,1.34) | 0.78 |
| **PSM** |  |  |  |  |  |  |  |  |
| post-icu statin | Ref |  | Ref |  | Ref |  | Ref |  |
| pre-icu statin | 2.64(1.53, 4.54) | <0.01 | 2.65(1.56, 4.52) | <0.01 | 0.99(0.64, 1.53) | 0.95 | 1.03(0.67,1.57) | 0.95 |
| **Fine-Gray** |  |  |  |  |  |  |  |  |
| post-icu statin | Ref |  | Ref |  | Ref |  | Ref |  |
| pre-icu statin | 2.91(1.27, 6.68) | 0.01 | 2.92(1.34, 6.35) | <0.01 | 1.21(0.79, 1.86) | 0.39 | 1.20(0.80, 1.82) | 0.38 |

HR, hazard ratio; CI, confidence interval; model 1: crude model, Age, Sex, Race, HR, SBP, DBP, MBP, CKD, LD, HTN, AF, COPD, DM, CHF, HLP, RBC, WBC, Platelet, Hemoglobin, Hematocrit, Aniongap, Bicarbonate; model 2: model 1, Bun, Calcium, Chloride, Creatinine, Glucose, Sodium, Potassium, INR, PT, PTT, SOFA, SAPSII, OASIS, GCS, CRRT, Ventilation, Antiplatelet drug, Anticoagulant drug, Vasoactive drug.

**Table 5. Association between duration of statin use (≥3, ≥5, and ≥7 Days) and all-cause ICU mortality based on Landmark analyses.**

| **Variable** | **HS-30-day mortality** | | **HS-90-day mortality** | | **IS-30-day mortality** | | **IS-90-day mortality** | |
| --- | --- | --- | --- | --- | --- | --- | --- | --- |
|  | HR 95%CI | P | HR 95%CI | P | HR 95%CI | P | HR 95%CI | P |
| No use | Ref |  | Ref |  | Ref |  | Ref |  |
| **Model1** |  |  |  |  |  |  |  |  |
| ≥3 days | 0.36(0.25,0.53) | <0.01 | 0.40(0.28,0.57) | <0.01 | 0.38(0.29,0.51) | <0.01 | 0.41(0.31,0.55) | <0.01 |
| ≥5 days | 0.46(0.29, 0.72) | <0.01 | 0.48(0.31, 0.74) | <0.01 | 0.41(0.29,0.58) | <0.01 | 0.44(0.32,0.61) | <0.01 |
| ≥7 days | 0.42(0.24, 0.73) | <0.01 | 0.45(0.27, 0.76) | <0.01 | 0.47(0.31,0.72) | <0.01 | 0.5(0.34,0.74) | <0.01 |
| **Model2** |  |  |  |  |  |  |  |  |
| ≥3 days | 0.48(0.32,0.72) | <0.01 | 0.52(0.35,0.76) | <0.01 | 0.45(0.33,0.62) | <0.01 | 0.50(0.37,0.68) | <0.01 |
| ≥5 days | 0.59(0.37, 0.95) | 0.03 | 0.61(0.38, 0.96) | 0.03 | 0.49(0.33,0.72) | <0.01 | 0.54(0.38,0.78) | 0.01 |
| ≥7 days | 0.51(0.28, 0.92) | 0.03 | 0.55(0.31, 0.96) | 0.04 | 0.6(0.37,0.95) | 0.03 | 0.65(0.42,1.01) | 0.06 |
| **PSM** |  |  |  |  |  |  |  |  |
| ≥3 days | 0.49(0.31, 0.78) | <0.01 | 0.57(0.36, 0.89) | 0.01 | 0.42(0.28, 0.63) | <0.01 | 0.5(0.34, 0.74) | <0.01 |
| ≥5 days | 0.5(0.28, 0.89) | 0.02 | 0.52(0.30, 0.92) | 0.02 | 0.52(0.32, 0.84) | 0.01 | 0.61(0.39, 0.95) | 0.03 |
| ≥7 days | 0.59(0.28, 1.23) | 0.16 | 0.58(0.28, 1.22) | 0.15 | 0.37(0.18, 0.75) | 0.01 | 0.49(0.26, 0.92) | 0.03 |
| **Fine–Gray** |  |  |  |  |  |  |  |  |
| ≥3 days | 0.59(0.38, 0.92) | 0.02 | 0.66(0.44, 1.01) | 0.05 | 0.49(0.34, 0.71) | <0.01 | 0.56(0.39, 0.79) | <0.01 |
| ≥5 days | 0.66(0.41, 1.09) | 0.11 | 0.66(0.40, 1.06) | 0.09 | 0.59(0.38, 0.91) | 0.02 | 0.67(0.45, 0.99) | 0.04 |
| ≥7 days | 0.57(0.31, 1.08) | 0.08 | 0.55(0.30, 1.04) | 0.07 | 0.67(0.39, 1.18) | 0.17 | 0.72(0.43, 1.18) | 0.19 |

HR, hazard ratio; CI, confidence interval; model 1: crude model, Age, Sex, Race, HR, SBP, DBP, MBP, CKD, LIV, HTN, AF, COPD, DM, CHF, HLP; RBC, WBC, Platelet, Hemoglobin, Hematocrit, Aniongap, Bicarbonate; model 2: model 1, Bun, Calcium, Chloride, Creatinine, Glucose, Sodium, Potassium, INR, PT, PTT, SOFA, SAPSII, OASIS, GCS, CRRT, Ventilation, Antiplatelet drug, Anticoagulant drug, Vasoactive drug.
